# Comparative Efficacy and Acceptability of Treatment Strategies for Antipsychotic-Induced Akathisia: A Systematic Review and Network Meta-analysis

**DOI:** 10.1093/schbul/sbae098

**Published:** 2024-06-13

**Authors:** Yuki Furukawa, Kota Imai, Yusuke Takahashi, Orestis Efthimiou, Stefan Leucht

**Affiliations:** Department of Neuropsychiatry, University of Tokyo Hospital, Tokyo, Japan; Pharmaceutical Department, University of Tokyo Hospital, Tokyo, Japan; Department of Neuropsychiatry, University of Tokyo Hospital, Tokyo, Japan; Department of Psychiatry, Tokyo Musashino Hospital, Tokyo, Japan; Institute of Primary Health Care (BIHAM), Faculty of Medicine, University of Bern, Bern, Switzerland; Institute of Social and Preventive Medicine (ISPM), Faculty of Medicine, University of Bern, Bern, Switzerland; Department of Psychiatry and Psychotherapy, School of Medicine and Health, Technical University of Munich, Munich, Germany

**Keywords:** Akathisia, antipsychotic, schizophrenia, network meta-analysis

## Abstract

**Background:**

Antipsychotics are the treatment of choice for schizophrenia, but they often induce akathisia. However, comparative efficacy of treatment strategies for akathisia remains unclear.

**Design:**

We performed a systematic review and network meta-analyses (PROSPERO CRD42023450720). We searched multiple databases on July 24, 2023. We included randomized clinical trials comparing 1 or more treatment strategies for antipsychotic-induced akathisia against each other or control conditions. We included adults with schizophrenia or other psychiatric disorders treated with antipsychotics. The primary outcome was akathisia severity at posttreatment. Secondary outcomes included akathisia response, all-cause dropout, psychotic symptoms, and long-term akathisia severity. We synthesized data in random effects frequentist network meta-analyses and assessed confidence in the evidence using CINeMA.

**Results:**

We identified 19 trials with 661 randomized participants (mean age 35.9 [standard deviation 12.0]; 36.7% [195 of 532] women). No trials examined dose reduction or switching of antipsychotics. Findings suggested 5-HT2A antagonists (*k* = 6, *n* = 108; standardized mean difference [SMD] −1.07 [95% confidence interval, −1.42; −0.71]) and beta-blockers (*k* = 8, *n* = 105; SMD −0.46 [−0.85; −0.07]) may improve akathisia severity, but confidence in the evidence was deemed low. We also found that benzodiazepines (*k* = 2, *n* = 13; SMD −1.62 [−2.64; −0.59]) and vitamin B6 (*k* = 3, *n* = 67; SMD −0.99 [−1.49; −0.50]) might also be beneficial, but confidence in the evidence was very low. Analyses of secondary outcomes did not provide additional insights.

**Conclusions:**

Our findings suggest that 5-HT2A antagonists, beta-blockers, and with a lesser certainty, benzodiazepines, and vitamin B6 might improve akathisia. Given the low to very low confidence in the evidence of add-on agents and the absence of evidence of their long-term efficacy, careful consideration of side effects is warranted. These recommendations are extremely preliminary and further trials are needed.

## Introduction

Antipsychotics are key drugs for treating schizophrenia, but almost 1 in 5 patients experience akathisia as a side effect.^1,2^ Akathisia is characterized by a subjective sense of inner restlessness, which sometimes urges patients to commit deleterious behaviors including suicide.^1,3^ Clinical practice guidelines recommend dose reduction, switching antipsychotic, and adjuvant medications.^4,5^ While the exact pathophysiological mechanism of akathisia remains unclear, the use of these add-on agents is based on hypotheses that antipsychotic-induced akathisia is caused by dopaminergic or serotonergic mechanisms. Anti-parkinsonian drugs, such as anticholinergics and antihistamines are used because akathisia is categorized as an extrapyramidal symptom and suspected to be related to dopaminergic neurotransmission. Beta-blockers may also exert their effects via the inhibitory effect of noradrenergic input to the ventral tegmental area, which, in turn, leads to increased dopamine neurotransmission.^6^ 5-HT2A antagonists and triptans may improve akathisia via serotonergic neurotransmission.^7,8^ Benzodiazepines and vitamin B6 are expected to be beneficial because they have been shown effective in some other movement disorders.^9,10^ Some adjuvant medications have been examined in a few randomized trials and meta-analyses.^11^ A recent network meta-analysis (NMA)^12^ on this topic found potential benefits of some adjuvant agents, but it did not include antipsychotic adjustments and it did not assess the overall confidence in the body of evidence according to CINeMA^13^ or GRADE.^14^ To examine the confidence in the evidence is a prerequisite of NMAs which must come on top of simple risk of bias assessment, in particular to examine whether direct and indirect evidence was enough in accordance to allow NMA. Although the procedure is time-intense, it is needed to make recommendations for practice.

In this study, we examined the comparative efficacy and acceptability of treatment strategies for antipsychotic-induced akathisia, including antipsychotic adjustments and adjuvant medications, using systematic review and NMA, and assessed the confidence in the evidence using CINeMA.^13^

## Methods

We followed the Preferred Reporting Items for Systematic reviews and Meta-Analyses (PRISMA) guideline extension for NMA.^15^ The protocol was prospectively registered in PROSPERO (CRD42023450720) and can be found in supplementary appendix 1.

### Data Sources

#### Criteria for Considering Studies for This Review.

We included randomized controlled trials comparing the treatment strategies for antipsychotic-induced akathisia against control conditions. In cross-over trials, we included only the first intervention period to avoid carry-over effects. We included trials on patients using antipsychotics of both sexes aged 18 years or older with schizophrenia or other psychiatric disorders.^16^ We included all drug-related interventions, such as dose reduction of the antipsychotic, switching the antipsychotic, and adjunctive medications such as 5-HT2A antagonists, anticholinergics, antihistamines, benzodiazepines, and beta-blockers (table 1). We excluded drugs in development and included only licensed drugs.

**Table 1.** Classification of the Interventions

Interventions	Drugs
Antipsychotic adjustments	
Dose reduction	Any antipsychotics
Switching	Any antipsychotics
Add-on agents	
5-HT2A antagonists	Mianserin, mirtazapine, and trazodone
Anticholinergics	Benztropine, biperiden, and trihexyphenidyl
Antihistamines	Cyproheptadine, diphenhydramine, and promethazine
Benzodiazepines	Clonazepam, diazepam, and lorazepam
Beta-blockers	Betaxolol, metoprolol, nadolol, and propranolol
Triptans	Zolmitriptan
Vitamin B	Vitamin B6

#### Search Methods for Identification of Studies.

We searched ClinicalTrials.gov, Cochrane Library (Cochrane Database of Systematic Reviews and Cochrane Central Register of Controlled Trials), Embase, MEDLINE, and PsycINFO via Ovid SP, PubMed, and WHO ICTRP on July 24, 2023 with no date, language, document type, and publication status restrictions. Search strategies were developed by a medical information scientist and are reported in supplementary appendix 2. We checked the reference lists of review articles for additional potentially eligible records.

### Data Collection and Analysis

#### Selection of Studies.

Pairs of 2 reviewers (Y.F., K.I., and Y.T.) independently screened titles and abstracts of all the potential studies. We retrieved full-text study reports of potentially eligible studies and pairs of 2 reviewers (Y.F., K.I., and Y.T.) independently screened them. We resolved any disagreement through discussion. We identified publications from the same trial so that each trial rather than each report was the unit of analysis in the review. We assessed the inter-rater reliability of the full-text screening decisions with Cohen’s κ and percentage agreement.

#### Data Items.

Pairs of 2 reviewers (Y.F., K.I., and Y.T.) extracted data from the included studies independently. We assessed risk of bias of the primary outcome of the included trials using the revised risk of bias tool by Cochrane^17^ in the 5 following domains: randomization process, deviations from intended interventions, missing outcome data, measurement of the outcome, and selection of reported results. Any disagreement was resolved through discussion. We measured the inter-rater reliability of the extracted data concerning the primary outcome with intraclass correlation, and the risk of bias assessment with weighted κ and percentage agreement.

#### Primary Outcome and Secondary Outcomes.

The primary outcome of interest in this study was treatment efficacy at treatment endpoint. As we prespecified in the protocol, we prioritized the Barnes Akathisia Scale (BAS) global scale and if not available the total score.^18^ If the BAS was not available, other validated scales were used. Secondary outcomes included efficacy using akathisia severity response (dichotomous), all-cause dropouts (as a proxy measure of treatment acceptability), psychotic symptom severity, and efficacy at long-term follow-up (continuous, longest follow-up between 1 and 12 months). Intention-to-treat analysis was prioritized whenever available. We used standardized mean differences (SMD) for continuous outcomes, because multiple scales were used, and odds ratio for dichotomous outcomes.^19^

### Statistical Analysis

We created a network diagram at the class level to visualize the available evidence. Classification of the interventions are described in table 1. Transitivity is a basic assumption behind NMA.^20^ To meaningfully combine the direct evidence from A vs C and B vs C studies to learn indirectly about the comparison A vs B, there should not be important differences in the distribution of the effect modifiers across treatment comparisons. We created box plots of trial and patient characteristics deemed to be possible effect modifiers (publication year, proportion of patients with antipsychotics likely to bring akathisia, and baseline severity) and visually examined whether they were similarly distributed across treatment comparisons. We checked consistency using global (design-by-treatment) and local (back-calculation) tests.^21,22^ We assessed possible reporting bias and small-study effects using contour-enhanced funnel plots of comparisons with 10 or more trials. Given the expected clinical and methodological heterogeneity of treatment effects among the studies, we conducted random-effects NMA. We visualized NMA results using placebo augmentation as reference and ordering treatments considering both the number of participants analyzed and *P*-scores for the primary outcome, which provide an overall ranking of treatments.^23^ We summarized the results in a table using a coloring scheme where colors denote beneficial or harmful effect and shading shows the strength of confidence in the evidence, which we assessed using the CINeMA approach.^13,24,25^ We also performed the pairwise created a league table showing NMA results together with direct evidence of pairwise meta-analyses. Using the weighted mean proportion of responders in the placebo arms as the control event rate, we translated the odds ratios into the experimental event rates to improve interpretability.^26^ Finally, we performed a series of prespecified sensitivity analyses on the primary outcome: (1) we performed individual drug-level NMA to examine the influence of lumping different drugs into classes; (2) we excluded trials with extremely different assessment time point to test the influence of including different endpoint (which was equal to excluding trials with intramuscular injections); and (3) we excluded trials with high overall risk of bias to test the influence of risk of bias.

We performed analyses in *R* version 4.2.3^27^ using *netmeta*^28^ and *meta*^29^ packages.

## Results

We identified 6290 records, assessed 127 full texts for eligibility and included 19 trials with 661 randomized participants in the meta-analysis. One trial^30^ reported only a dichotomous efficacy outcome, and therefore was not included in the primary analysis (supplementary appendix 2). We read the reports carefully to ensure that each trial was used as the unit of analysis, not each report. The inter-rater reliability of judgements for full-text screening was good, with κ of 0.67 (95% CI, 0.53–0.81) and percentage agreement of 85%. Supplementary appendix lists^31^ the included and excluded trials with reasons for exclusion (supplementary appendix 2).

Typical participants were males in their thirties (mean age 35.9 [SD 12.0]; 36.7 % [195 of 532] were females). The included trials were small (mean number of participants randomized = 35 [SD 27]). The mean follow-up time was 6.0 (SD 4.3) days. No trial had long-term follow-up longer than 1 month. No trial examined dose reduction or switching the antipsychotics. Publication year ranged from 1988 to 2020. Majority of the trials were conducted in the Middle East (11 out of 19). Table 2 tabulates the characteristics of included trials. The overall risk of bias according to the Cochrane’s revised risk of bias tool was low in none of the trials, some concerns in 7 (37%, 7 out of 19) and high in 12 (63%, 12 out of 19). The inter-rater reliability for the overall risk of bias was slight, with a weighted κ = 0.15 (95% CI, 0.00–0.65) and percentage agreement 55%. Inter-rater reliability of extracted primary outcomes was almost perfect, with an intraclass correlation (ICC) of 0.94 (95% CI, 0.88–0.98).

**Table 2.** Characteristics of Included Trials

Study	Country	Multicentered	Akathisia Definition	Age, Years Mean (SD)	Antipsychotics	Number Randomized	Drug	Overall Risk of Bias
Aoba et al^30^	Japan	Yes	BARS	N.A.	N.A.	103	Cartelol, placebo	H
Avital et al^8^	Israel	No	DSM-IV-TR	36.4 (10.4)	Mixed	33	Propranolol, zolmitriptan	H
Baskak et al^32^	Turkey	No	DSM-IV	33.2 (11.1)	Mixed	31	Biperiden, placebo	SC
Dumon et al^31^	France	No	N.A.	39.9 (13.2)	Typical	18	Betaxolol, propranolol	SC
Fischel et al^33^	Israel	No	DSM-IV	33.4 (9.3)	Typical	30	Cyproheptadine, propranolol	H
Irwin et al^34^	USA	No	BARS	36.8 (11.1)	Typical	11	Propranolol, placebo	SC
Kramer et al^35^	USA	No	N.A.	36.5 (8.8)	Typical	20	Propranolol, placebo	H
Kramer et al^36^	USA	No	N.A.	36.5 (9.0)	Typical	12	Propranolol, placebo	H
Kutcher et al^9^	Canada	No	Others^a^	18.5 (1.5)	Typical	15	Clonazepam, placebo	H
Lerner et al^37^	Israel	Yes	DSM-IV	42.5 (14.7)	Mixed	20	Vitamin B6, placebo	SC
Midownik et al^38^	Israel	Yes	DSM-IV	41.8 (13.3)	Mixed	60	Mianserin, placebo	SC
Poyurovsky et al^39^	Israel	No	DSM-IV	29.7 (9.1)	Typical	30	Vitamin B6, placebo	H
Poyurovsky et al^40^	Israel	No	DSM-IV	30.5 (10.3)	Typical	26	Mirtazapine, placebo	H
Poyurovsky et al^41^	Israel	No	DSM-IV	34.2 (10.8)	Typical	90	Mirtazapine, propranolol, placebo	H
Pujalte et al^42^	France	No	BARS	31.5 (7.5)	Typical	12	Clonazepam, placebo	H
Shams-Alizadeh et al^10^	Iran	No	N.A.	35.3 (13.1)	Mixed	60	Propranolol, vitamin B6	H
Shams-Alizadeh et al^43^	Iran	No	BARS	42.0 (11.9)	Mixed	52	Trazodone, placebo	SC
Stryjer et al^44^	Israel	No	DSM-IV	43.0 (10.6)	NA	13	Trazodone, placebo	SC
Wells et al^45^	USA	No	Others^b^	33.8 (6.3)	Typical	25	Nadolol, placebo	H

*Note:* BARS, Barnes Akathisia Rating Scale; DSM-IV, Diagnostic and Statistical Manual of Mental Disorders, fourth edition; DSM-IV-TR, Diagnostic and Statistical Manual of Mental Disorders, fourth edition, text revised; H, high risk of bias; N.A., not applicable; SC, some concerns; SD, standard deviation.

^a^Akathisia subscale of the Chouinard extrapyramidal symptom rating scale.

^b^Akathisia subscale of Extrapyramidal Symptom Rating Scale.

**Fig. 1.**
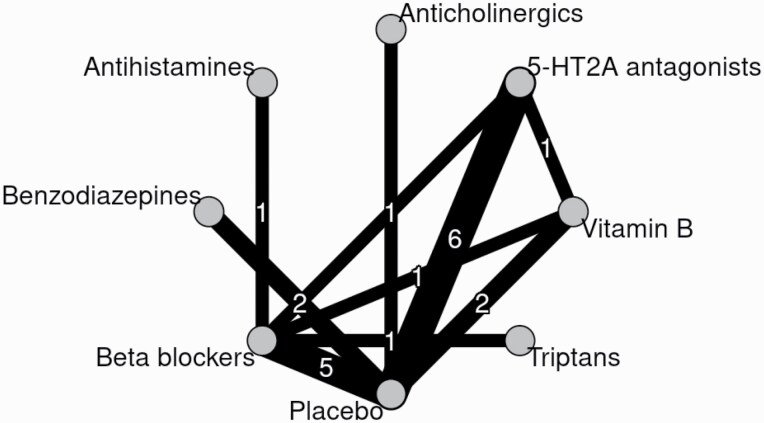
Network diagram.

**Fig. 2.**
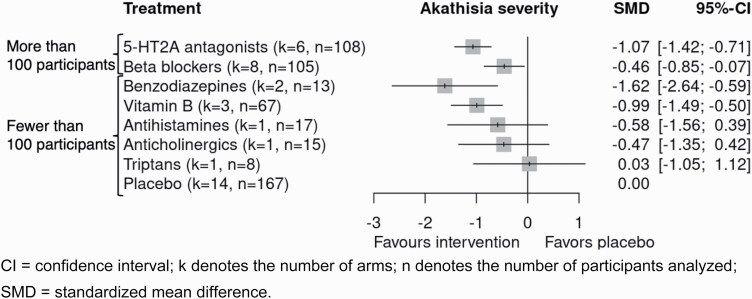
Results from network meta-analysis, for the primary outcome.

The network for the primary outcome at the class level was well-connected (figure 1). Assessment of transitivity (supplementary appendix 3) found that potential effect modifiers were evenly distributed across comparisons, except for the publication year of beta-blockers and benzodiazepine, which were mainly examined earlier in the 1990s. The global (design-by-treatment) test did not show evidence of inconsistency (*P* = .66). The local (back-calculation) method did not find evidence of disagreement between direct and indirect comparisons (assessed in 6 comparisons). The limited number of trials precluded an evaluation of publication bias and small-study effects using funnel plots. Figure 2 and table 3 show the results of the class level NMA and supplementary appendix 3 shows the results of the pairwise meta-analyses and the league table. Supplementary appendix 3 shows the result of CINeMA.^13^ We found that 5-HT2 antagonists (*k* = 6, *n* = 108; SMD −1.07 [95% confidence interval, −1.42 to −0.71]) and beta-blockers (*k* = 8, *n* = 105; SMD −0.46 [−0.85 to −0.07]) may be beneficial, but the confidence in the evidence was low according to CINeMA, mainly due to the high overall risk of bias in the original trials. We also found that benzodiazepines (*k* = 2, *n* = 13; SMD −1.62 [−2.64 to −0.59]) and vitamin B6 (*k* = 3, *n* = 67; SMD −0.99 [−1.49 to −0.50]) may be superior to placebo in improving akathisia; confidence in the evidence was, however, very low. Results of the secondary (binary) efficacy outcome were in line with the primary analysis (results in table 3). Dropout for any reason had very wide confidence intervals and did not provide any evidence on the comparative risk of add-on agents (results in table 3). Prespecified sensitivity analyses were in line with the primary analysis (supplementary appendix 3). We estimated the weighted average proportion of responders in placebo arms to be 16%. Applying the estimated odds ratios of the binary response NMA to this proportion, we estimated that 5-HT2A antagonists may lead to response in 64% (95% CI, 47%–78%) and beta-blockers in 39% (95% CI, 26%–53%) of the patients.^26^

**Table 3.** Results From Network Meta-analysis, All Outcomes

	Primary Outcome	Secondary Outcomes		
	Akathisia Severity	Akathisia Response	Dropout	Psychotic Symptoms
Trials, No.	17	18	10	10
Heterogeneity				
tau^2^	0.07	0.00	0.00	0.04
*I*^2^ (95% CI)	28.5% (0.0%; 63.0%)	0.0% (0.0%; 55.0%)	0.0% (0.0%; 79.2%)	22% (0.0%; 65.1%)
Treatments				
Effect measures	SMD (95% CI)	OR (95% CI)	OR (95% CI)	SMD (95% CI)
More than 100 participants were included for the primary outcome				
5-HT2A antagonists	−1.07 (−1.42; −0.71)	9.58 (4.85; 18.9)	0.56 (0.23; 1.35)	0.09 (−0.28; 0.45)
Beta-blockers	−0.46 (−0.85; −0.07)	3.38 (1.92; 5.95)	0.99 (0.39; 2.49)	−0.23 (−0.77; 0.31)
Fewer than 100 participants were included for the primary outcome				
Benzodiazepines	−1.62 (−2.64; −0.59)	41.2 (3.97; 428)	3.00 (0.37; 11.81)	—
Vitamin B6	−0.99 (−1.49; −0.50)	7.20 (3.06; 17.0)	0.99 (0.17; 5.78)	−0.21 (−0.75; 0.34)
Antihistamines	−0.58 (−1.56; 0.39)	3.79 (0.78; 18.4)	2.12 (0.07; 64.2)	1.10 (0.04; 2.16)
Anticholinergics	−0.47 (−1.35; 0.42)	1.56 (0.36; 6.69)	3.00 (0.11; 79.5)	−0.39 (−1.22; 0.45)
Triptans	0.03 (−1.05; 1.12)	1.58 (0.24; 10.5)	—	−0.52 (−1.63; 0.58)

*Note*: CI, confidence interval; OR, odds ratio; SMD, standardized mean difference. Light gray indicates treatments being more effective than placebo for beneficial outcomes (akathisia severity and akathisia response), and not more harmful for harmful outcomes (dropout and psychotic symptoms) based on low to very low certainty of evidence according to CINeMA. Dark gray indicates treatments being not better than placebo for beneficial outcomes and more harmful for harmful outcomes based on low to very low certainty of evidence according to CINeMA.

## Discussion

This systematic review and meta-analyses found 19 trials with 661 participants (17 trials with 500 participants for the primary analysis), for treatments of antipsychotic-induced akathisia. We sought for trials examining dose reduction and switching of antipsychotics, but we found no eligible trials. There was some evidence suggesting that 5-HT2A antagonists and beta-blockers may improve akathisia severity, but the confidence in the evidence was low, mainly owing to the high risk of bias of the original studies. We also found evidence suggesting that benzodiazepines and vitamin B6 may be also beneficial, but the confidence was very low. These finding are based on short-term follow-up only and no trials examined whether the effects persist on the long-term.

We come up with different conclusions than the recent drug-level NMA.^12^ Our study has several advantages. First, with the help of a medical information scientist, we conducted a more comprehensive systematic literature review and found more trials (*k* = 19 vs *k* = 15). The differences in the assessment of clonazepam and biperiden seem to stem from the difference in the included trials. As for clonazepam, we included Kutcher et al^9^ but they excluded it because it included some participants younger than 18. We decided to include it because the majority of the participants were 18 years or older and the average age was older than 18. As for biperiden, they included Friis et al,^46^ although they stated in the protocol that they would include only the first intervention period to avoid carry-over effects and the paper reported only the pooled results of a cross-over trial. We excluded it in accordance with our prespecified protocol. It should also be noted that both trials are very small (*n* = 15 for both trials), and that this discrepancy reflects the very low robustness of evidence, which should be clearly stated. Second, we evaluated the risk of bias of each trial according to the official guidance. There is concern that the assessment of the recent study was lenient. For example, they considered 13 studies to be at low risk of bias in the randomization process. However, none reported if the allocation was properly concealed. According to the official guidance document, this should lead to some concerns at best. Third, to assess the confidence in the evidence is an essential component of NMA, but it was not applied in the previous NMA. We assessed confidence in the body of evidence with the established framework CINeMA,^13^ and found the certainty of evidence was low at best. Given the low to very low certainty of evidence, it is essential to emphasize the limitation of the results drawn, and the limited conclusions which can be made for practice.

Dose reduction and switching the antipsychotics are the first-line recommendations in clinical practice guidelines.^4,5^ Although we did not find any trials directly examining these strategies, these strategies remain reasonable; a dose-response meta-analysis of antipsychotics found that higher doses impose a greater risk of akathisia,^47^ and an NMA^16^ showed that different antipsychotics carry different risks of akathisia.^16^ Albeit the lack of direct evidence, these strategies should still be considered, given the low to very low confidence in the evidence of add-on agents and the absence of evidence of their long-term efficacy. In contrast to our NMA on pharmacological add-on strategies based on only 19 RCTs and 661 participants, the dose-response meta-analysis,^47^ included 98 RCTs and 34 225 participants, and the one^16^ on akathisia risk of various antipsychotics 116 RCTs and 25 783 participants, thus a much more solid evidence-base.

Clinical practice guideline recommendations for add-on pharmacotherapies vary from guideline to guideline. Some lists all the drugs based on the positive results of a few randomized controlled trials,^11,12^ while some discourages the use of concomitant agents based on the very limited body of evidence.^5^ A moderate approach is to make a weak recommendation for add-on agents that have at least a little evidence and that are widely used in clinical practice.^4^ This study is the first to address the issue with the most systematic and established way of evidence synthesis and confidence assessment. Given the low to very low certainty in the evidence, potential short-term benefit should be weighed carefully with possible side effects. 5-HT2A antagonists (mianserin, trazodone, and mirtazapine) induce somnolence and weight gain.^48,49^ Beta-blockers may lead not only to cardiovascular adverse events such as bradycardia and arrhythmia, but also dizziness and fatigue.^50^ Benzodiazepines are known for side effects, such as residual sedation^51^ dependence/withdrawal,^52^ and falls.^53^ Even though vitamin B6 is water-soluble, there is still a risk of overdose, which may induce polyneuropathy.^54^

The strengths of our study are as follows. First, we used NMA, which allowed us to include 5 comparisons examining an active intervention to another. These comparisons were excluded in the conventional pairwise meta-analyses comparing an intervention against placebo. This improved the precision of effect estimates and enabled the evaluation of comparative efficacy. Second, we assessed the confidence in the evidence using CINeMA, which enabled us to present the results in a balanced manner.

Our study has several limitations. The most notable limitation is the limited number and size of included trials. Even for the most examined agents, there were less than ten trials, with the total number randomized to the agents being only around 100. The median sample size was 26, thus most trials were very small. It is well known from meta-epidemiological studies that small trials often exaggerate effects.^55^ This may explain that for several agents SMD were around 1, which is rare in schizophrenia. For example, it seems unlikely that vitamin B6, the treatment recommended most by Gerolymos et al^12^, produces a SMD of 0.99. Indeed, according to CINeMA the confidence in its evidence was very low. Second, many of the trials were old and many methodological advances in clinical trials have been made since then. The overall risks of bias were judged to be high in almost two-thirds of the trials, with some concerns in the remaining ones. Third, many trials took place before the second-generation antipsychotics became the mainstream approach to treating schizophrenia, and the results may not be completely applicable to the current clinical practice.^4^

## Conclusion

We found a possible short-term benefit of adding 5-HT2A antagonists, beta-blockers (low confidence in the evidence), benzodiazepines, and vitamin B6 (very low confidence in the evidence). However, the body of evidence is extremely preliminary and lacks long-term evaluation. Adjuvant medications may only be considered when it is difficult to reduce the dose of or switch the antipsychotic used, and with careful consideration of side effects.

## Supplementary Material

Supplementary material is available at https://academic.oup.com/schizophreniabulletin/.

sbae098_suppl_Supplementary_Appendix

## Data Availability

Data and code used for analyses are available on GitHub (https://github.com/ykfrkw/AIA_NMA).

## Patient and Public Involvement

There was no patient or public involvement in the development of this manuscript.

## Registration

This protocol is prospectively registered in PROSPERO (CRD42023450720). This research was prospectively registered (#2023178NIe), Ethical Committee, Faculty of Medicine, The University of Tokyo.
